# γ-Alumina-supported Pt_17_ cluster: controlled loading, geometrical structure, and size-specific catalytic activity for carbon monoxide and propylene oxidation†

**DOI:** 10.1039/c9na00579j

**Published:** 2019-12-03

**Authors:** Yuichi Negishi, Nobuyuki Shimizu, Kanako Funai, Ryo Kaneko, Kosuke Wakamatsu, Atsuya Harasawa, Sakiat Hossain, Manfred E. Schuster, Dogan Ozkaya, Wataru Kurashige, Tokuhisa Kawawaki, Seiji Yamazoe, Shuhei Nagaoka

**Affiliations:** Department of Applied Chemistry, Faculty of Science, Tokyo University of Science 1-3 Kagurazaka, Shinjuku-ku Tokyo 162-8601 Japan negishi@rs.kagu.tus.ac.jp; Photocatalysis International Research Center, Tokyo University of Science 2641 Yamazaki, Noda Chiba 278-8510 Japan; Johnson Matthey Technology Centre Blounts Court, Sonning Common Reading RG4 9NH UK; Johnson Matthey Japan, G.K. 5123-3, Kitsuregawa, Sakura Tochigi 329-1492 Japan Shuhei.Nagaoka@mattheyasia.com; Department of Chemistry, Graduate School of Science, Tokyo Metropolitan University 1-1 Minami-Osawa, Hachioji-shi Tokyo 192-0397 Japan yamazoe@tmu.ac.jp

## Abstract

Although Pt is extensively used as a catalyst to purify automotive exhaust gas, it is desirable to reduce Pt consumption through size reduction because Pt is a rare element and an expensive noble metal. In this study, we successfully loaded a Pt_17_ cluster on γ-alumina (γ-Al_2_O_3_) (Pt_17_/γ-Al_2_O_3_) using [Pt_17_(CO)_12_(PPh_3_)_8_]Cl_*n*_ (*n* = 1, 2) as a precursor. In addition, we demonstrated that Pt is not present in the form of an oxide in Pt_17_/γ-Al_2_O_3_ but instead has a framework structure as a metal cluster. Moreover, we revealed that Pt_17_/γ-Al_2_O_3_ exhibits higher catalytic activity for carbon monoxide and propylene oxidation than γ-Al_2_O_3_-supported larger Pt nanoparticles (Pt_NP_/γ-Al_2_O_3_) prepared using the conventional impregnation method. Recently, our group discovered a simple method for synthesizing the precursor [Pt_17_(CO)_12_(PPh_3_)_8_]Cl_*n*_. Furthermore, Pt_17_ is a Pt cluster within the size range associated with high catalytic activity. By combining our established synthesis and loading methods, other groups can conduct further research on Pt_17_/γ-Al_2_O_3_ to explore its catalytic activities in greater depth.

## Abstract 2

A Pt_17_ cluster was precisely loaded on γ-alumina and the resulting Pt_17_/γ-Al_2_O_3_ exhibited high catalytic activity for CO and C_3_H_6_ oxidation.

## Graphical abstract

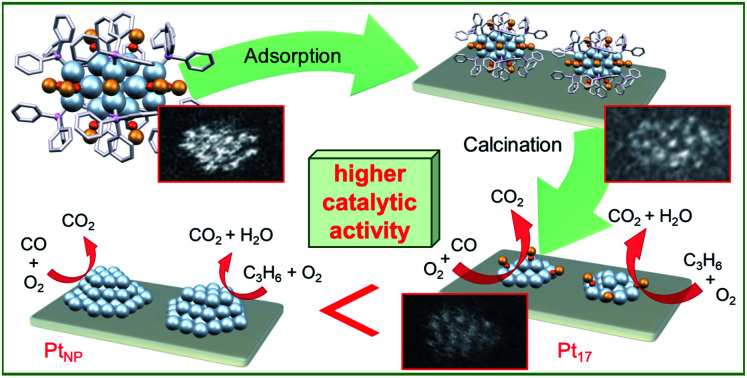

## Introduction

With rapid advances in science and technology, automobiles have become indispensable in our daily lives. Because Pt can catalytically eliminate harmful substances contained in exhaust gas, this metal along with Rh and Pd are extensively used to treat exhaust gas.^1^ However, Pt is a rare element and an expensive precious metal. Therefore, it is essential to reduce the amount of consumed Pt.

Many attempts have been made to develop catalysts without Pt. However, previous studies have implied that the activity and durability of Pt are superior to those of non-precious metals. To reduce the amount of Pt consumed while taking advantage of its characteristic features, it is essential to improve its activity and performance per unit weight of the catalyst. The size reduction of Pt nanoparticles/clusters (hereinafter: Pt_*n*_ clusters) increases the proportion of surface atoms^2,3^ and enables the creation of new geometrical/electronic structures;^4–10^ thus, this approach can efficiently reduce Pt consumption.^11–13^

Meanwhile, the geometrical/electronic structures and chemical properties of Pt_*n*_ clusters in the fine size range vary considerably depending on the number of constituent atoms.^14^ Therefore, it is important to load Pt_*n*_ clusters with a controlled number of constituent atoms on a substrate to create a highly active supported Pt catalyst using fine Pt_*n*_ clusters while elucidating the catalytic activity and performance of the clusters. Using a vacuum device with a mass selector,^2,6,10,15–19^ it is possible to load controlled Pt_*n*_ clusters onto a substrate. In fact, magnesium-oxide-supported Pt_*n*_ clusters (Pt_*n*_/MgO; *n* = 8–20) and titanium-dioxide-supported Pt_*n*_/TiO_2_ (*n* = 4, 7–10, 15) have been prepared with precisely controlled numbers of Pt atoms using these types of experiments. These studies also revealed that fine supported Pt_*n*_ clusters exhibit high catalytic activity for the oxidation of carbon monoxide (CO).^2,19^ However, for the practical use of supported Pt catalysts, issues remain regarding device manufacturing costs and loading efficiency for the preparation of supported Pt_*n*_ clusters using such vacuum equipment.

**Fig. 1:**
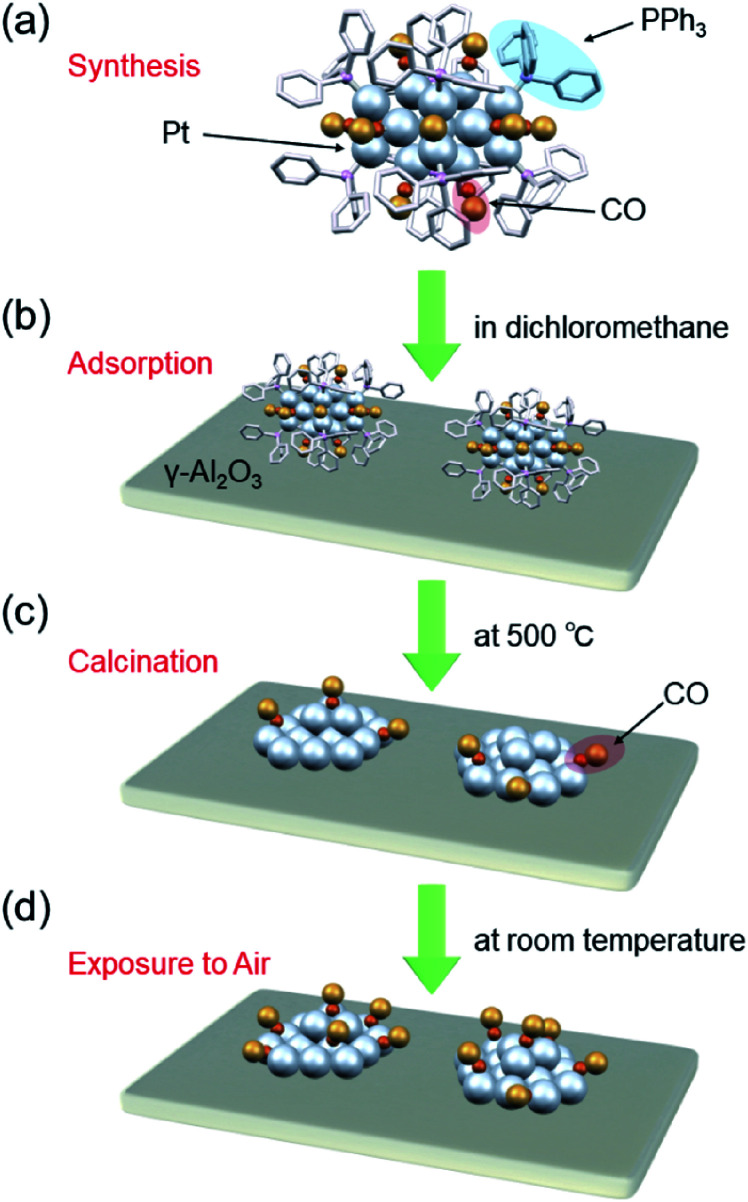
Schematic of preparation of Pt_17_/γ-Al_2_O_3_: (a) precise synthesis of [Pt_17_(CO)_12_(PPh_3_)_8_]Cl_*n*_, (b) adsorption of [Pt_17_(CO)_12_(PPh_3_)_8_]Cl_*n*_ on γ-Al_2_O_3_ (Pt_17_(CO)_12_(PPh_3_)_8_/γ-Al_2_O_3_), (c) calcination of ligands while maintaining the cluster size (Pt_17_/γ-Al_2_O_3_), and (d) exposure of Pt_17_/γ-Al_2_O_3_ to air.

Recently, it has become possible to precisely synthesize various noble-metal and noble-metal-alloy clusters with atomic accuracy.^20–49^ Pt_*n*_ clusters can be synthesized with atomic accuracy using CO as a ligand or two types of ligand, CO and phosphine.^48^ In addition, Pt_*n*_ clusters can be precisely synthesized using special dendrimers as templates.^50,51^ When these Pt_*n*_ clusters are adsorbed on a substrate followed by the removal of the ligands, Pt_*n*_ clusters with a controlled number of constituent atoms can be loaded on the substrate without the issues of device construction cost and loading efficiency (Fig. 1). However, currently, there are few examples of controlled loading of Pt_*n*_ clusters on a substrate using this approach. For the synthesis of the former ligand-protected Pt_*n*_ clusters, it is essential to carry out the reaction under a CO atmosphere. For the synthesis of the latter dendrimer-protected Pt_*n*_ clusters, a special dendrimer synthesis technique is needed. Therefore, few research groups are capable of conducting these precise syntheses, and fundamental and applied research on fine supported Pt_*n*_ clusters is currently limited.

**Fig. 2:**
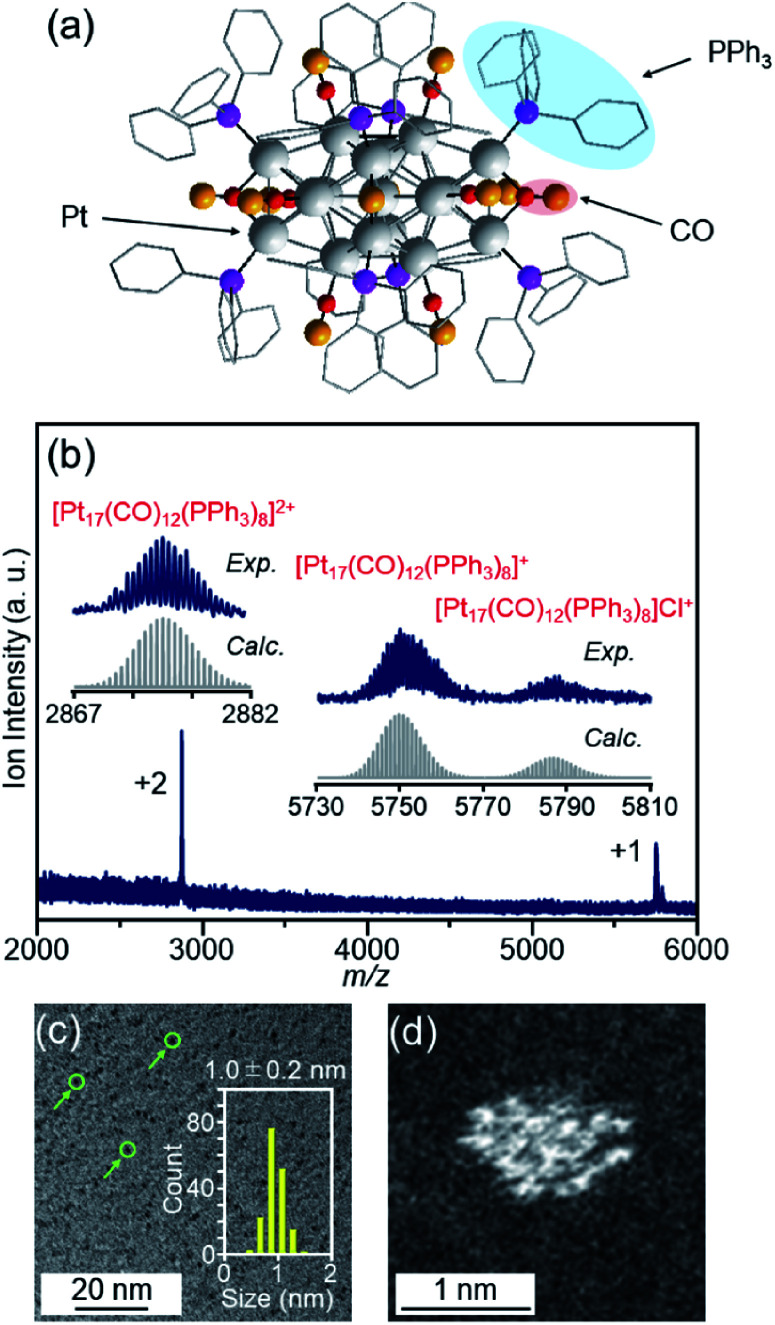
(a) Geometrical structure of [Pt_17_(CO)_12_(PPh_3_)_8_]^+^ determined by single-crystal X-ray crystallography. The geometrical structure (a) was reproduced from ref. 51. (b) Positive-ion ESI mass spectrum of [Pt_17_(CO)_12_(PPh_3_)_8_]Cl_*n*_ synthesized in this work. The spectrum indicates that both [Pt_17_(CO)_12_(PPh_3_)_8_]Cl_*n*_ (*n* = 1, 2) are contained in this sample, similar to the previous work.^52^ (c) TEM and (d) HAADF-STEM images of [Pt_17_(CO)_12_(PPh_3_)_8_]Cl_*n*_. In (c), the green circles indicate the particles and the histogram estimated from the TEM images is also provided. Copyright 2017 American Chemical Society.

We recently discovered a very simple method for synthesizing Pt_17_ clusters protected with CO and triphenylphosphine (PPh_3_) ([Pt_17_(CO)_12_(PPh_3_)_8_]Cl_*n*_; *n* = 1, 2; Fig. 2(a)).^52^ In our synthesis method, first, the Pt_*n*_(CO)_*m*_(PPh_3_)_*l*_ cluster, which is mainly composed of [Pt_17_(CO)_12_(PPh_3_)_8_]Cl_*n*_, is prepared by mixing the reagents and heating the solvent in the atmosphere. Then, the main product, [Pt_17_(CO)_12_(PPh_3_)_8_]Cl_*n*_, is separated from the obtained mixture with high purity using the difference in solubility. This method does not require special synthesis equipment or dendrimer synthesis techniques. If we could establish the loading method of the Pt_17_ cluster using [Pt_17_(CO)_12_(PPh_3_)_8_]Cl_*n*_ as a precursor, many research groups would be able to obtain fine supported Pt_17_ catalysts.

In this study, the following three goals were addressed with the final objective of using supported Pt_*n*_ clusters as catalysts to treat automotive exhaust gas: (i) establishment of a precise loading method of Pt_17_ on γ-alumina (γ-Al_2_O_3_); (ii) structural analysis of the obtained Pt_17_/γ-Al_2_O_3_; and (iii) evaluation of the catalytic activity of Pt_17_/γ-Al_2_O_3_ against the oxidation reaction of CO and propylene (C_3_H_6_). As a result, we successfully determined the conditions for loading Pt_17_ on γ-Al_2_O_3_ while preserving the size of Pt_17_ (Fig. 1). We observed that the supported Pt_17_ is not present in the form of an oxide^53^ but has a framework structure as a metal cluster in the obtained Pt_17_/γ-Al_2_O_3_. Furthermore, Pt_17_/γ-Al_2_O_3_ exhibited higher catalytic activity against the oxidation of CO and C_3_H_6_ than γ-Al_2_O_3_-supported larger Pt nanoparticles (Pt_NP_/γ-Al_2_O_3_) prepared using the conventional impregnation method.

## Results and discussion

### Loading of the Pt_17_ cluster on γ-Al_2_O_3_

[Pt_17_(CO)_12_(PPh_3_)_8_]Cl_*n*_ (Fig. 2(a)) was synthesized using our previously reported method (see the Experimental section).^52^ In this method, Pt_*x*_(CO)_*y*_(PPh_3_)_*z*_ clusters containing both CO and PPh_3_ were synthesized by mixing the reagents and heating the solvent in the atmosphere. Because CO, which is one of the ligands, can be generated by the oxidation of ethylene glycol,^54^ this method does not require equipment for preparing a CO atmosphere. Specifically, an ethylene glycol solution containing a Pt salt (H_2_PtCl_6_) and sodium hydroxide (NaOH) was heated at 120 °C in the atmosphere, and then PPh_3_ was added at room temperature to obtain Pt_*x*_(CO)_*y*_(PPh_3_)_*z*_ clusters. [Pt_17_(CO)_12_(PPh_3_)_8_]Cl_*n*_ was separated from the obtained mixture using the difference in solubility in the solvent (see the Experimental section; Scheme S1†). The electrospray ionization (ESI) (Fig. 2(b)) and matrix-assisted laser desorption/ionization (MALDI) (Fig. S1†) mass spectra of the product indicate that the product contained high-purity [Pt_17_(CO)_12_(PPh_3_)_8_]Cl_*n*_. The transmission electron microscopy (TEM) (Fig. 2(c)) and high-angle annular dark field-scanning transmission electron microscopy (HAADF-STEM) results (Fig. 2(d)) of the product were also consistent with the results of mass spectrometry (Fig. 2(b) and S1†).

The resulting [Pt_17_(CO)_12_(PPh_3_)_8_]Cl_*n*_ was first adsorbed onto γ-Al_2_O_3_ (Fig. 1(b)). In an aprotic solvent, the metal oxide has a permanent dipole moment on the surface.^55^ As reported by Tsukuda *et al.*, when this surface comes into contact with a metal cluster containing a functional group with a high dielectric constant (*e.g.*, a phenyl group) in the ligand, an induced dipole moment is generated in the ligand layer, and the metal clusters are adsorbed on the surface of the metal oxide *via* a dipole–induced dipole interaction.^56^ In the [Pt_17_(CO)_12_(PPh_3_)_8_]Cl_*n*_ used in this study, the ligand layer contained a large amount of phenyl groups, and [Pt_17_(CO)_12_(PPh_3_)_8_]Cl_*n*_ was thus adsorbed onto γ-Al_2_O_3_*via* a dipole–induced dipole interaction. Dichloromethane was used as the aprotic solvent. The concentration of [Pt_17_(CO)_12_(PPh_3_)_8_]Cl_*n*_ in solution was carefully controlled by inductively coupled plasma mass spectrometry (ICP-MS) such that the weight of Pt_17_ was 0.15 wt% relative to γ-Al_2_O_3_. The solution changed from brown to colorless and transparent after 2 h of stirring, indicating that practically all of the [Pt_17_(CO)_12_(PPh_3_)_8_]Cl_*n*_ was adsorbed onto γ-Al_2_O_3_ (Pt_17_(CO)_12_(PPh_3_)_8_/γ-Al_2_O_3_).

**Fig. 3:**
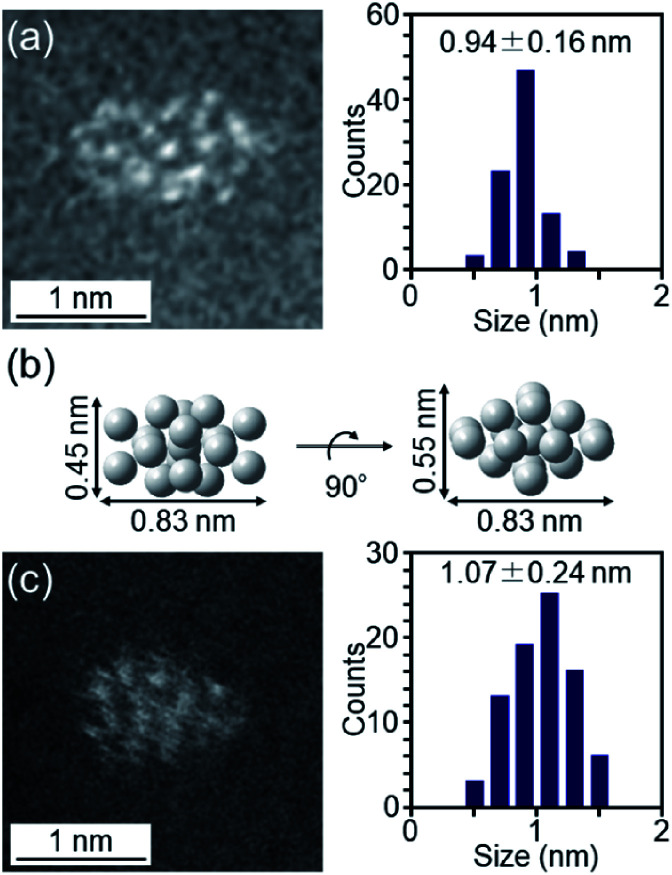
(a) HAADF-STEM image of Pt_17_(CO)_12_(PPh_3_)_8_/γ-Al_2_O_3_. (b) Pt_17_-core structure (two-angle views) of [Pt_17_(CO)_12_(PPh_3_)_8_]Cl_*n*_ (Fig. 2(a)) on the same scale as (a) and (c). (c) HAADF-STEM image of Pt_17_/γ-Al_2_O_3_. In (a) and (c), the histogram and average diameters estimated from the various HAADF-STEM images (Fig. S3 and S6†) are also included.

**Fig. 4:**
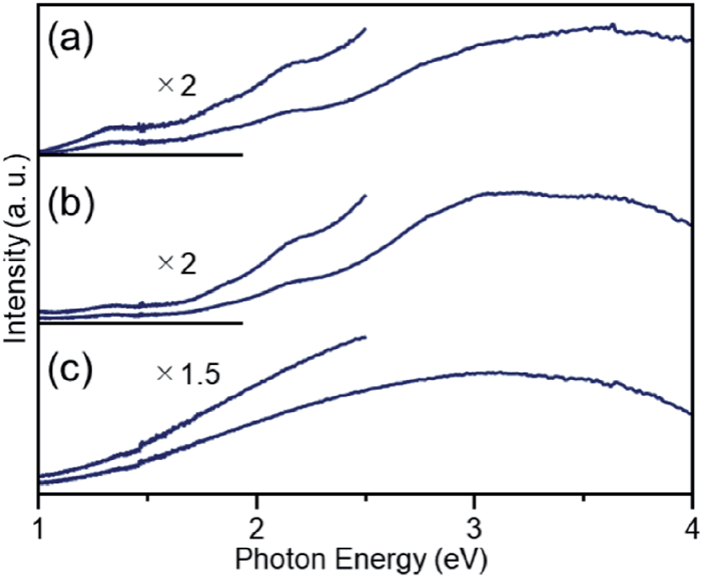
DR spectra of (a) [Pt_17_(CO)_12_(PPh_3_)_8_]Cl_*n*_, (b) Pt_17_(CO)_12_(PPh_3_)_8_/γ-Al_2_O_3_, and (c) Pt_17_/γ-Al_2_O_3_. For (b) and (c), the spectra were obtained by the subtraction of γ-Al_2_O_3_. The DR spectral profile of [Pt_17_(CO)_12_(PPh_3_)_8_]Cl_*n*_ (a) differs slightly from that reported in our previous work.^52^ This difference likely results from the different ratios of the two kinds of charged clusters, [Pt_17_(CO)_12_(PPh_3_)_8_]Cl_*n*_ (*n* = 1 or 2), in the two studies.

The particle diameter of Pt_17_(CO)_12_(PPh_3_)_8_/γ-Al_2_O_3_ obtained using this approach was estimated by HAADF-STEM measurement. In the HAADF-STEM image in Fig. 3(a), particles (0.94 ± 0.16 nm) with sizes similar to that of [Pt_17_(CO)_12_(PPh_3_)_8_]Cl_*n*_ (1.0 ± 0.2 nm; Fig. 2(d) and S2†) were observed with a narrow distribution (Fig. S3†). The HAADF-STEM image of Pt_17_(CO)_12_(PPh_3_)_8_/γ-Al_2_O_3_ (Fig. 3(a)) shows that it contained many particles with shapes similar to that of the Pt_17_ core of the precursor [Pt_17_(CO)_12_(PPh_3_)_8_]Cl_*n*_ (Fig. 3(b)). In the diffuse reflection (DR) spectrum of Pt_17_(CO)_12_(PPh_3_)_8_/γ-Al_2_O_3_, a peak structure similar to that of [Pt_17_(CO)_12_(PPh_3_)_8_]Cl_*n*_ was observed (Fig. 4(a) and (b)). These results indicate that aggregation of Pt_17_(CO)_12_(PPh_3_)_8_ hardly occurred during the adsorption process and that Pt_17_(CO)_12_(PPh_3_)_8_ after adsorption retained the geometrical/electronic structure of the precursor [Pt_17_(CO)_12_(PPh_3_)_8_]Cl_*n*_.

Then, the PPh_3_ ligands were removed from Pt_17_(CO)_12_(PPh_3_)_8_/γ-Al_2_O_3_ by calcination^57–61^ (Fig. 1(c)). Based on thermogravimetric mass spectrometry (TG-MS) analysis, a temperature of approximately 400 °C is required for PPh_3_ removal (Fig. S4†).^62^ Thus, PPh_3_ was removed from the Pt_17_ cluster by calcination at 500 °C. In the DR spectra of the sample after calcination (Fig. 4(c)), the peaks of [Pt_17_(CO)_12_(PPh_3_)_8_]Cl_*n*_ (Fig. 4(a)) and Pt_17_(CO)_12_(PPh_3_)_8_/γ-Al_2_O_3_ (Fig. 4(b)) were not observed. In the X-ray photoelectron spectrum after calcination (Fig. S5†), the P 2p peak was not observed. In the HAADF-STEM image of the sample after calcination (Fig. 3(c)), particles (1.07 ± 0.24 nm) with sizes similar to that of Pt_17_(CO)_12_(PPh_3_)_8_/γ-Al_2_O_3_ (0.94 ± 0.16 nm; Fig. 3(a)) were observed with a narrow distribution (Fig. S6†). These results indicate that the PPh_3_ ligands were removed from the cluster by calcination and that Pt_17_ did not aggregate during this process. Pt forms a relatively strong bond with O compared with other noble metals (318.4 ± 6.7 kJ mol^−1^ for Pt–O *vs.* 223 ± 21.1 kJ mol^−1^ for Au–O).^63^ Furthermore, as γ-Al_2_O_3_ has a complicated structure in which Al atoms are arranged octahedrally or tetrahedrally, cationic sites are present because of the surface defects in γ-Al_2_O_3_.^64^ Pt clusters could be strongly immobilized on γ-Al_2_O_3_ by the interaction between Pt atoms and these cationic sites.^53^ For these reasons, it is considered that Pt_17_ did not aggregate on γ-Al_2_O_3_ during calcination. To confirm the weight of Pt loaded on γ-Al_2_O_3_, Pt_17_/γ-Al_2_O_3_ was mixed with aqua regia, and the amount of dissolved Pt was measured using ICP optical emission spectroscopy (ICP-OES). The results confirmed that 0.15 wt% Pt was actually loaded on γ-Al_2_O_3_. The results of temperature-programmed reaction measurements indicate that the surface of the supported Pt_17_ was covered by CO at normal temperature (Fig. 1(d), S7 and S8†). This result is most likely due to the existence of uneliminated CO (Fig. 1(c)) as well as the adsorption of CO from the atmosphere.

### Structural analysis of Pt_17_/γ-Al_2_O_3_

To understand deeply the charge state and geometrical structure of the obtained Pt_17_/γ-Al_2_O_3_, the charge state and geometry of the Pt_17_ cluster were investigated using X-ray absorption fine structure (XAFS) analysis.

**Fig. 5:**
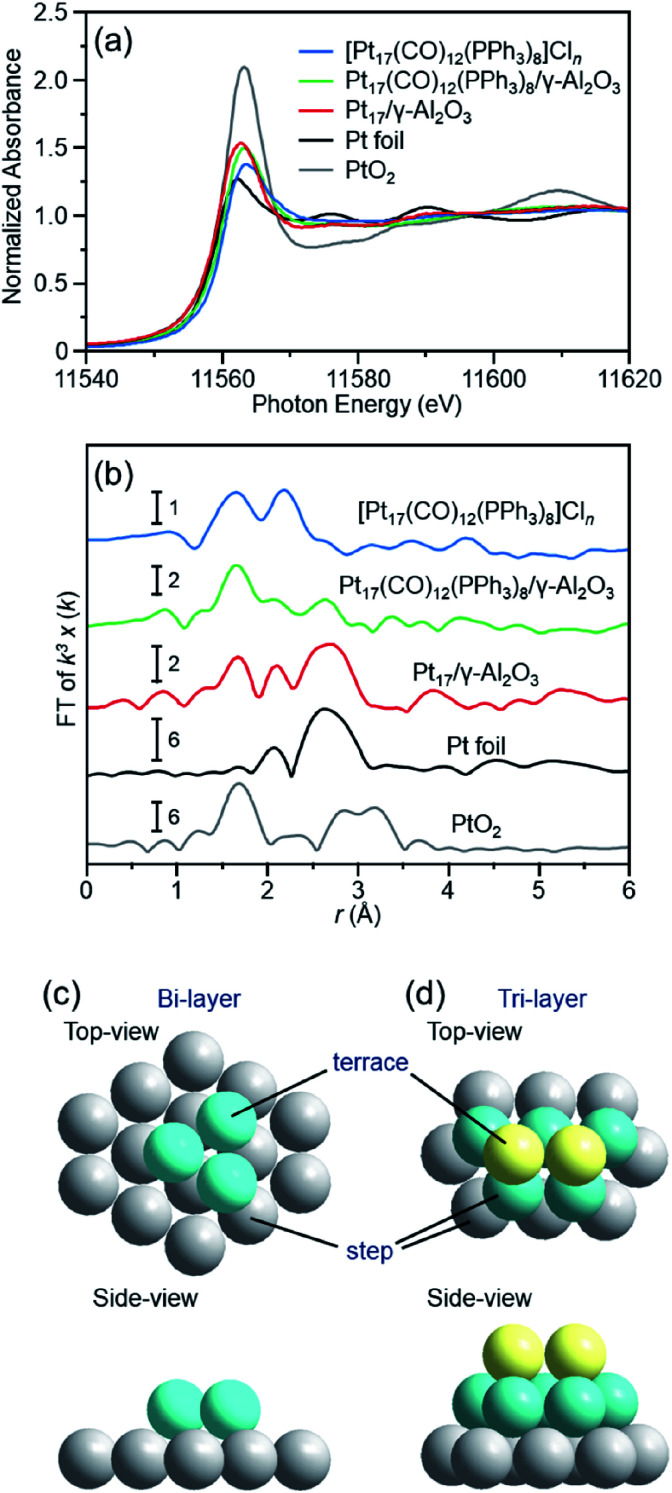
(a) Pt L_3_-edge XANES and (b) Pt L_3_-edge FT-EXAFS spectra of [Pt_17_(CO)_12_(PPh_3_)_8_]Cl_*n*_, Pt_17_(CO)_12_(PPh_3_)_8_/γ-Al_2_O_3_, and Pt_17_/γ-Al_2_O_3_ together with those of Pt foil and PtO_2_ for comparison. (c) and (d) two proposed structures for Pt_17_ on γ-Al_2_O_3_, which were estimated based on the HAADF-STEM images (Fig. 3(c) and S6†) of Pt_17_/γ-Al_2_O_3_ (Fig. S11†). In (b), the peak at ∼2.3 Å in the spectrum of Pt foil is attributed to the satellite peak of the Pt–Pt bond. In (c) and (d), both top and side views are shown.

The Pt L_3_-edge X-ray absorption near-edge structure (XANES) spectra of [Pt_17_(CO)_12_(PPh_3_)_8_]Cl_*n*_, Pt_17_(CO)_12_(PPh_3_)_8_/γ-Al_2_O_3_, and Pt_17_/γ-Al_2_O_3_ are shown in Fig. 5(a) together with those of Pt foil and PtO_2_ for comparison. The white-line intensities of [Pt_17_(CO)_12_(PPh_3_)_8_]Cl_*n*_, Pt_17_(CO)_12_(PPh_3_)_8_/γ-Al_2_O_3_, and Pt_17_/γ-Al_2_O_3_ are similar to that of Pt foil and very different from that of PtO_2_. This result indicates that Pt is not present as an oxide in Pt_17_.^53^ Among the three samples, the white-line intensity increases in the order of [Pt_17_(CO)_12_(PPh_3_)_8_]Cl_*n*_ → Pt_17_(CO)_12_(PPh_3_)_8_/γ-Al_2_O_3_ → Pt_17_/γ-Al_2_O_3_. This result indicates that the number of holes in the d orbital of Pt_17_ increases, namely the electron density of Pt_17_ decreases, in this order.

Fig. 5(b) shows the Pt L_3_-edge Fourier-transform extended X-ray absorption fine structure (FT-EXAFS) spectra of [Pt_17_(CO)_12_(PPh_3_)_8_]Cl_*n*_, Pt_17_(CO)_12_(PPh_3_)_8_/γ-Al_2_O_3_, and Pt_17_/γ-Al_2_O_3_ (Tables S1–S3 and Fig. S9†). In the FT-EXAFS spectrum of [Pt_17_(CO)_12_(PPh_3_)_8_]Cl_*n*_, the peaks attributed to the Pt–C and Pt–P bonds appear at ∼1.7 and ∼2.3 Å, respectively. For Pt_17_(CO)_12_(PPh_3_)_8_/γ-Al_2_O_3_, the intensity of the peak at ∼1.7 Å increased and that at ∼2.3 Å decreased, and the peak attributed to the Pt–Pt bond appeared at ∼2.8 Å. As described above, there is no significant difference in the optical absorption between [Pt_17_(CO)_12_(PPh_3_)_8_]Cl_*n*_ and Pt_17_(CO)_12_(PPh_3_)_8_/γ-Al_2_O_3_ (Fig. 4(a) and (b)). Therefore, it is assumed that the Pt_17_ cluster maintains the metal-core structure as a whole even during adsorption (Fig. 3(a) and (b)). However, the FT-EXAFS spectrum indicates that the adsorption causes a slight change in the structure of the ligand layer that covers Pt_17_. For the appearance of the Pt–Pt bond in the spectrum, a plausible explanation is that the variation in the Pt–Pt bond length (Fig. S10†) decreases or the fluctuation of the Pt–Pt bond decreases^65^ with adsorption on the substrate. The decrease in the electron density of the d orbital of Pt_17_ (Fig. 5(a)) caused by adsorption can also likely be attributed to the structural change of the ligand layer. In the spectrum of Pt_17_/γ-Al_2_O_3_ after calcination, a peak at ∼2.8 Å clearly appears, and its satellite peak (in the FT-EXAFS spectrum of the Pt foil in Fig. 5(b)) is also observed at ∼2.3 Å.^66^ This result indicates that the variation in the Pt–Pt bond length and/or the fluctuation of the Pt–Pt bond further decrease with the PPh_3_ removal and/or the structural change of the Pt_17_ cluster from the icosahedral-based structure (Fig. 2(a)) to the structure shown in Fig. 5(c) and (d) (see below). In this spectrum, a peak was also observed at ∼1.7 Å. As described above, the surface of supported Pt_17_ is covered by CO at normal temperature. The peak at ∼1.7 Å is attributed to the generated Pt–C or Pt–O bond at the Pt_17_/γ-Al_2_O_3_ interface.

Thus, it was observed that Pt does not form an oxide^53^ and that Pt_17_ has a framework structure like a metal cluster in Pt_17_/γ-Al_2_O_3_. Based on the HAADF-STEM image, the supported Pt_17_ is assumed to have a bi-layer^2^ or tri-layer structure, as shown in Fig. 5(c), (d) and S11.† Previous studies have suggested that CO and O_2_ are activated on the terrace Pt and step Pt, respectively, during the oxidation reaction of CO.^2,3,18^Fig. 5(c) and (d) show that most of the terrace Pt is located near the step Pt in Pt_17_/γ-Al_2_O_3_. Thus, the reaction of CO and O_2_, *i.e.*, the oxidation of CO, is expected to proceed effectively over Pt_17_/γ-Al_2_O_3_.

### Catalytic activity of Pt_17_/γ-Al_2_O_3_ against the oxidation reaction of CO and C_3_H_6_

**Fig. 6:**
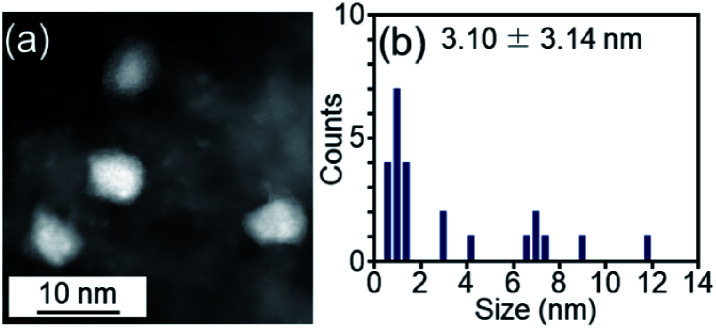
(a) Representative HAADF-STEM image and (b) histogram estimated from various HAADF-STEM images of Pt_NP_/γ-Al_2_O_3_ prepared for comparison.

We examined the catalytic activity of the obtained Pt_17_/γ-Al_2_O_3_. Industrially used supported Pt catalysts are frequently prepared by the impregnation method. Therefore, in this study, Pt_NP_/γ-Al_2_O_3_, in which 0.15 wt% Pt was loaded by the impregnation method, was used as a comparative Pt catalyst. The amount of Pt was confirmed by mixing Pt_NP_/γ-Al_2_O_3_ with aqua regia and measuring the concentration of dissolved Pt using ICP-OES. The HAADF-STEM image shown in Fig. 6 indicates that Pt_NP_/γ-Al_2_O_3_ has an average particle size of 3.10 ± 3.14 nm.

The obtained Pt_17_/γ-Al_2_O_3_ and Pt_NP_/γ-Al_2_O_3_ were examined for their catalytic activity against the oxidation of CO and C_3_H_6_, which are the main components in automobile gas.^1^ In an actual automobile, the catalysts are coated on a honeycomb substrate made of cordierite ceramic. Thus, in this study, Pt_17_/γ-Al_2_O_3_ and Pt_NP_/γ-Al_2_O_3_ were coated on a honeycomb substrate to evaluate their catalytic performance in a state similar to the actual vehicle mounting conditions (Scheme S2†).

#### CO oxidation reaction

**Scheme 1:**
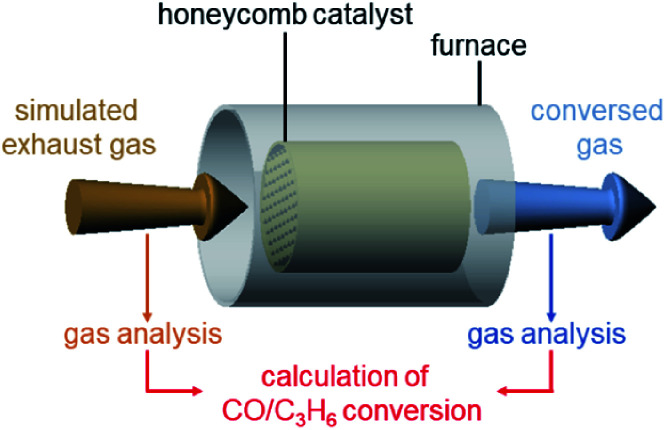
Schematic illustration of the estimation of CO and C_3_H_6_ conversions over Pt_17_/γ-Al_2_O_3_ or Pt_NP_/γ-Al_2_O_3_ coated on a cordierite honeycomb substrate.

An engine exhaust-gas-measuring device was used to determine the catalytic activity. In the experiments, a gas mixture consisting of 1% CO, 0.5% O_2_, and 98.5% N_2_ was circulated over the honeycomb substrate at a space velocity of 50 000 L h^−1^ while increasing the temperature of the honeycomb substrate to 400 °C at a rate of 20 °C min^−1^ (Table S4†). The conversion ratio of CO over the catalyst was estimated by evaluating the components of the mixed gas before and after circulation using an exhaust gas analyzer (Scheme 1).

**Fig. 7:**
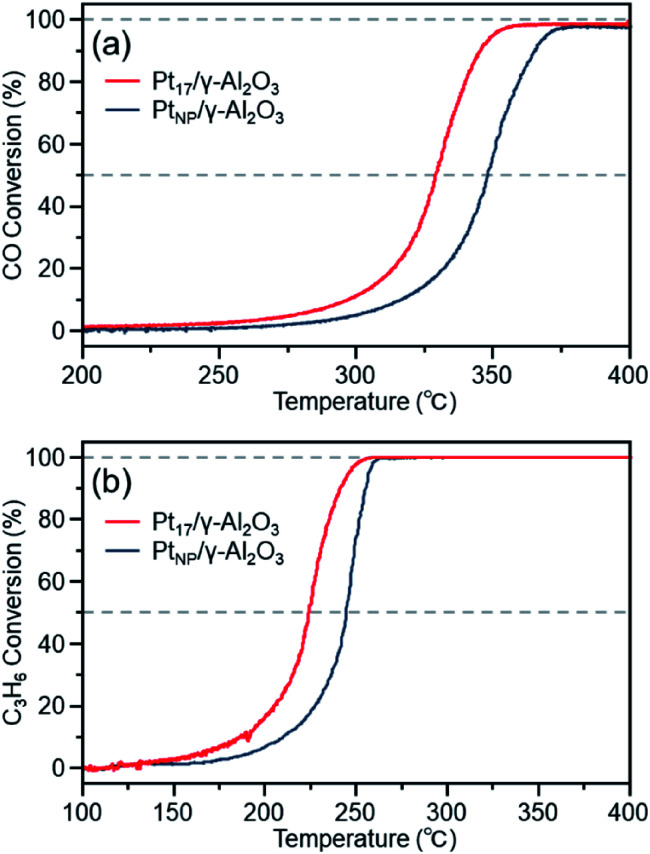
(a) CO and (b) C_3_H_6_ conversions over Pt_17_/γ-Al_2_O_3_ or Pt_NP_/γ-Al_2_O_3_.

Fig. 7(a) shows the CO conversion for each catalyst (Pt_17_/γ-Al_2_O_3_ or Pt_NP_/γ-Al_2_O_3_) estimated using this approach. When Pt_NP_/γ-Al_2_O_3_ was used as the catalyst, the catalytic activity started to appear at approximately 270 °C, and the conversion reached 50% at approximately 350 °C (light-off temperature) and nearly 100% at approximately 370 °C. However, when Pt_17_/γ-Al_2_O_3_ was used, the catalytic activity started to manifest at approximately 240 °C, and the conversion reached 50% at approximately 330 °C and almost 100% at approximately 350 °C. These results indicate that Pt_17_/γ-Al_2_O_3_ exhibits higher catalytic activity at each temperature than Pt_NP_/γ-Al_2_O_3_ and thus that Pt_17_/γ-Al_2_O_3_ can treat CO at lower temperatures than Pt_NP_/γ-Al_2_O_3_. Currently, the issue of enhanced activity of exhaust-gas-treating catalysts at low temperatures must be overcome with the spread of vehicles that frequently stop and restart their engines (*e.g.*, hybrid vehicles).^1^ These results strongly suggest that Pt_17_/γ-Al_2_O_3_ could be used as an exhaust gas-treating catalyst to overcome this issue.

The higher activity of Pt_17_/γ-Al_2_O_3_ than Pt_NP_/γ-Al_2_O_3_ is considered to be associated with their respective geometrical structures.^67^ Although the geometrical structures of Pt_17_/γ-Al_2_O_3_ and Pt_NP_/γ-Al_2_O_3_ before the reaction experiments are shown in Fig. 5 and 6, these geometrical structures should change as the catalytic reaction progresses and have not yet been elucidated.^68^ However, there should be more combinations composed of the terrace and step Pt in Pt_17_/γ-Al_2_O_3_ than in Pt_NP_/γ-Al_2_O_3_ (Fig. 5(c) and (d)). These geometrical effects appear to make the reaction between CO and O_2_ more likely to occur in Pt_17_/γ-Al_2_O_3_, resulting in higher CO conversion of Pt_17_/γ-Al_2_O_3_ at any temperature. In addition, Pt_17_ in Pt_17_/γ-Al_2_O_3_ should be more susceptible to the fluctuation of the geometrical/electronic structure than Pt_NP_ in Pt_NP_/γ-Al_2_O_3_. The ease of fluctuation of their geometrical/electronic structures may also contribute to the high activity of Pt_17_/γ-Al_2_O_3_.^9^ Furthermore, CO adsorbed on fine Pt_*n*_ supported clusters generally has a longer C–O bond than that adsorbed on the larger Pt_*n*_ supported nanoparticles, which promotes the oxidation reaction.^69^ In addition to the geometric factors, it is assumed that such a difference in CO activation caused by the difference in electronic states between the two supported clusters also contributes to the high activity of Pt_17_/γ-Al_2_O_3_.

#### C_3_H_6_ oxidation reaction

In this experiment, a mixed gas containing 200 ppm C_3_H_6_, 0.5% O_2_, and ∼99.5% N_2_ was circulated at a space velocity of 50 000 L h^−1^ while increasing the temperature of the honeycomb substrate to 400 °C at a rate of 20 °C min^−1^ (Table S4†). The conversion ratio of C_3_H_6_ over the catalyst was estimated by evaluating the components of the mixed gas before and after circulation using an exhaust gas analyzer (Scheme 1).

Fig. 7(b) shows the C_3_H_6_ conversion for each catalyst (Pt_17_/γ-Al_2_O_3_ or Pt_NP_/γ-Al_2_O_3_) estimated using this approach. When Pt_NP_/γ-Al_2_O_3_ was used as a catalyst, the catalytic activity started to manifest at approximately 160 °C, and the conversion reached 50% at approximately 245 °C and nearly 100% at approximately 260 °C. However, when Pt_17_/γ-Al_2_O_3_ was used, the catalytic activity started to manifest at approximately 130 °C, and the conversion reached 50% at approximately 225 °C and nearly 100% at approximately 250 °C. These results indicate that Pt_17_/γ-Al_2_O_3_ exhibits higher catalytic activity at each temperature than Pt_NP_/γ-Al_2_O_3_ for oxidizing C_3_H_6_. Currently, the mechanism of C_3_H_6_ oxidation is not as well understood as that of CO oxidation.^70^ Therefore, it is difficult to discuss the origin of the difference between the two activities. However, there should be a large difference between Pt_17_/γ-Al_2_O_3_ and Pt_NP_/γ-Al_2_O_3_ in the number of surface Pt atoms that can participate in the reaction. It appears that this factor is responsible for the difference in activity of the two types of catalysts.

#### Durability

We also investigated the durability of Pt_17_/γ-Al_2_O_3_ and Pt_NP_/γ-Al_2_O_3_. In this experiment, the catalysts were experimentally aged to simulate the deteriorated state of the catalysts caused by engine operation of an automobile. First, the honeycomb substrate was exposed to an oxidizing atmosphere (a gas mixture of 3% O_2_, 10% water vapor (H_2_O), and 87% N_2_) at 1000 °C for 3 min. Then, the honeycomb substrate was exposed to a reducing atmosphere (a gas mixture of 3% H_2_, 3% CO, 10% H_2_O, and 84% N_2_) at 1000 °C for 3 min (Table S5†). These operations were repeated for 4 h. Then, the CO or C_3_H_6_ conversion of Pt_17_/γ-Al_2_O_3_ and Pt_NP_/γ-Al_2_O_3_ was estimated using the method described above (Scheme 1).

**Fig. 8:**
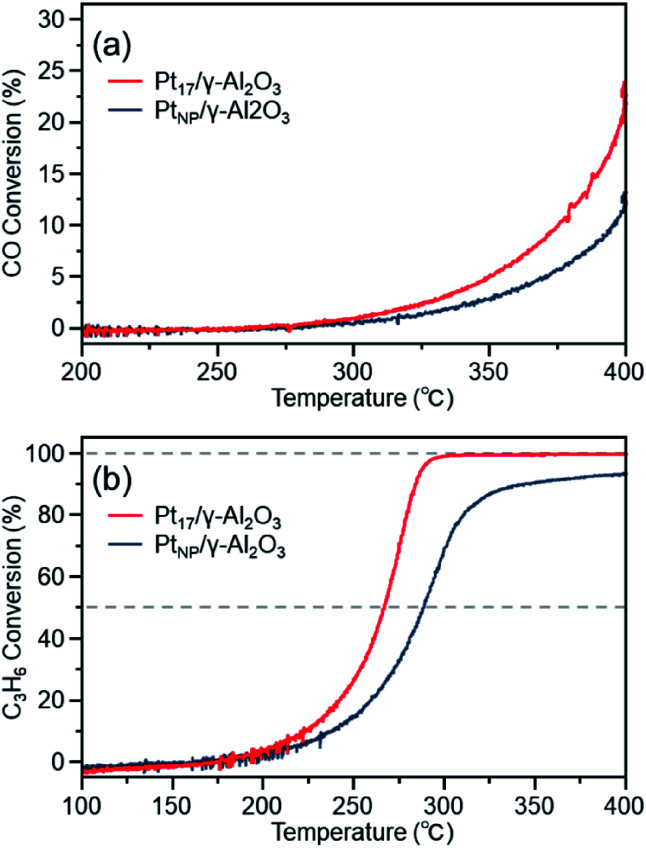
(a) CO and (b) C_3_H_6_ conversions over Pt_17_/γ-Al_2_O_3_ or Pt_NP_/γ-Al_2_O_3_ after the aging treatment.

Fig. 8(a) shows the CO conversion of each catalyst (Pt_17_/γ-Al_2_O_3_ or Pt_NP_/γ-Al_2_O_3_) after the aging treatment. The CO conversion rate decreased significantly for both catalysts compared with that before the aging treatment (Fig. 7(a)). A similar phenomenon was observed for the C_3_H_6_ conversion. These results indicate that the previously described procedure results in deterioration of the performance of both catalysts. However, comparing the conversion over Pt_17_/γ-Al_2_O_3_ and Pt_NP_/γ-Al_2_O_3_, the use of Pt_17_/γ-Al_2_O_3_ resulted in higher conversion than the use of Pt_NP_/γ-Al_2_O_3_ for both reactions. This result indicates that Pt_17_/γ-Al_2_O_3_ exhibits higher activity than Pt_NP_/γ-Al_2_O_3_ even after the aging treatment.

The decrease in activity after aging is generally induced by the aggregation of the supported Pt catalyst.^12,71^ In fact, the aggregation of the Pt catalyst was observed after the aging treatment for both Pt_17_/γ-Al_2_O_3_ and Pt_NP_/γ-Al_2_O_3_ (Fig. S12†). However, the average particle size of Pt_17_/γ-Al_2_O_3_ and Pt_NP_/γ-Al_2_O_3_ after the aging treatment was 25.3 ± 19.4 and 77.5 ± 29.9 nm, respectively. Thus, the average particle size of the former was smaller than that of the latter even after the aging treatment. It is considered that because the original Pt_17_/γ-Al_2_O_3_ had a smaller particle size than the original Pt_NP_/γ-Al_2_O_3_, Pt_17_/γ-Al_2_O_3_ had a smaller average particle size than Pt_NP_/γ-Al_2_O_3_ after aggregation, resulting in its higher activity even after the aging treatment.

## Conclusions

In this study, we successfully developed a method for producing Pt_17_/γ-Al_2_O_3_ using [Pt_17_(CO)_12_(PPh_3_)_8_]Cl_*n*_ as a precursor. Characterization of the obtained Pt_17_/γ-Al_2_O_3_ revealed that Pt_17_ is not present in the form of an oxide but has a framework structure as a metal cluster. Furthermore, it was determined that Pt_17_/γ-Al_2_O_3_ exhibits better catalytic activity for CO and C_3_H_6_ oxidation as well as better durability than Pt_NP_/γ-Al_2_O_3_ prepared using the conventional impregnation method. The precursor [Pt_17_(CO)_12_(PPh_3_)_8_]Cl_*n*_ can be isolated with atomic precision only by mixing the reagents, heating the solvent in the atmosphere, and operating a simple separation process. The supported Pt_17_ is a Pt_*n*_ cluster within the size range associated with high catalytic activity.^18^ It is expected that by using the loading method established in this study, many research groups can conduct further investigations on Pt_17_/γ-Al_2_O_3_ to obtain a deeper understanding of this catalyst and find new ways of using Pt_17_/γ-Al_2_O_3_ and Pt_17_ supported on other oxides. However, for practical use of the catalyst, it is necessary to investigate the catalytic activity and durability under the actual operating conditions of the exhaust gas mixing ratio.^18,72–74^ In addition, the loading weight also needs to be increased to that used under the actual operating conditions (Fig. S13†). We are currently attempting the measurements under such conditions with collaborations between academia and industry.

## Experimental

### Chemicals

All the chemicals used in this study were commercially obtained and used without further purification. Hydrogen hexachloroplatinate hexahydrate (H_2_PtCl_6_·6H_2_O) was purchased from Tanaka Kikinzoku. Sodium hydroxide (NaOH), triphenylphosphine (PPh_3_), platinum (Pt) standard solution (1000 ppm), and bismuth (Bi) standard solution (100 ppm) were obtained from FUJIFILM Wako Pure Chemical Co. Acetone, acetonitrile, dichloromethane, ethylene glycol, hydrochloric acid, methanol, nitric acid, and toluene were sourced from Kanto Chemical Co., Inc. Pt nitrate (Pt(NO_3_)_4_) was obtained from Johnson Matthey. *trans*-2-[3-(4-*tert*-Butylphenyl)-2-methyl-2-propenylidene]malononitrile (DCTB) was purchased from Tokyo Chemical Industry. Cordierite honeycomb (25.4 mm *φ* × 50 mm *L*, 400 cells per in^2^) and γ-Al_2_O_3_ (Puralox SCFa-160, Sasol) were obtained from NGK Insulators, Ltd. Pure Milli-Q water (18.2 MΩ cm) was generated using a Merck Millipore Direct 3 UV system.

### Synthesis of [Pt_17_(CO)_12_(PPh_3_)_8_]Cl_*n*_

[Pt_17_(CO)_12_(PPh_3_)_8_]Cl_*n*_ was synthesized using the method reported in our previous paper^52^ with a slight modification (Scheme S1†). First, H_2_PtCl_6_·6H_2_O (0.10 mmol) and NaOH (2.2 mmol) were dissolved in ethylene glycol (25 mL). NaOH was used to control the pH of the solution and thereby suppress the particle size obtained by the polyol reduction.^54,75^ Then, the mixture was heated at 120 °C for 10 min to reduce the Pt ions and produce CO catalyzed by Pt ions. The color of the solution changed from yellow to dark brown. After cooling to room temperature (25 °C), acetone (10 mL) containing PPh_3_ (0.52 g, 2.0 mmol) was added to this solution at once. After several minutes, toluene (∼20 mL) and water (∼20 mL) were added to the reaction solution. The Pt clusters including Pt_17_(CO)_12_(PPh_3_)_8_ were transferred into the organic phase. Then, the organic phase was separated from the water phase and dried with a rotary evaporator. The dried product was washed with water and then methanol to eliminate ethylene glycol and excess PPh_3_. At this stage, the product was still a mixture of clusters of several sizes. The product was dried, and the by-products were then washed with a mixture of acetonitrile/toluene (1 : 1).

### Preparation of catalysts

#### Pt_17_/γ-Al_2_O_3_

First, the obtained [Pt_17_(CO)_12_(PPh_3_)_8_]Cl_*n*_ was adsorbed on γ-Al_2_O_3_ by mixing a dichloromethane solution containing 2.6 mg of [Pt_17_(CO)_12_(PPh_3_)_8_]Cl_*n*_ with a dichloromethane solution containing 1.00 g γ-Al_2_O_3_ for 2 h at room temperature. The total volume of the solution was fixed at 250 mL, and the mixing ratio of [Pt_17_(CO)_12_(PPh_3_)_8_]Cl_*n*_ to γ-Al_2_O_3_ was fixed at 0.15 wt% Pt. The amount of Pt in the solution was confirmed by ICP-MS analysis of the supernatant solution. After mixing for 2 h, the solution became colorless, which indicates that almost all the [Pt_17_(CO)_12_(PPh_3_)_8_]Cl_*n*_ were adsorbed on γ-Al_2_O_3_. Then, the obtained Pt_17_(CO)_12_(PPh_3_)_8_/γ-Al_2_O_3_ was calcined under reduced pressure (>1.0 × 10^−1^ Pa) to remove the PPh_3_ ligands (Fig. S4†). The furnace temperature was increased at a rate of 5 °C min^−1^ and then maintained at 500 °C for 20 min.

#### Pt_NP_/γ-Al_2_O_3_

An aqueous solution containing Pt(NO_3_)_4_ with 0.15 wt% Pt was impregnated onto the γ-Al_2_O_3_ support followed by drying at 120 °C for 2 h and calcination in air at 600 °C for 2 h to yield Pt_NP_/γ-Al_2_O_3_.

#### Monolithic honeycomb catalyst

Before the catalytic activity tests, monolithic honeycomb catalysts were prepared by coating a slurry, which was prepared from the Pt catalyst powder (Pt_17_/γ-Al_2_O_3_ or Pt_NP_/γ-Al_2_O_3_), an inorganic binder, and water, onto a cordierite honeycomb substrate followed by drying at 120 °C for 1 h and subsequent calcination at 500 °C for 2 h (Scheme S2†). The calcined honeycomb catalysts contained approximately 60 g L^−1^ of the coated catalyst powders.

### Characterization

ESI mass spectrometry was performed using a reflectron time-of-flight mass spectrometer (Bruker, micrOTOF II). In these measurements, a cluster solution with a concentration of ∼10 μg mL^−1^ in dichloromethane was electrosprayed at a flow rate of 180 μL h^−1^.

MALDI mass spectra were collected using a spiral time-of-flight mass spectrometer (JEOL, JMS-S3000) with a semiconductor laser. DCTB^76^ was used as the MALDI matrix (cluster : matrix = 1 : 1000).

TEM images were recorded with a JEM-2100 electron microscope (JEOL) operating at 200 kV, typically using a magnification of 600 000.

HAADF-STEM images were recorded using a JEOL ARM200CFE fitted with an aberration corrector. The catalyst powders of Pt_17_/γ-Al_2_O_3_ or Pt_NP_/γ-Al_2_O_3_ were ground between two glass slides and dusted onto a holey carbon-coated Cu TEM grid.

DR spectra were acquired at ambient temperature using a V-670 spectrometer (JASCO). The wavelength-dependent optical data (*I*(*w*)) were converted to the energy-dependent data (*I*(*E*)) using the following equation that conserved the integrated spectral areas: *I*(*E*) = *I*(*w*)/|∂*E*/∂*w*| ∝ *I*(*w*) × *w*^2^.

ICP-MS was performed using an Agilent 7500c spectrometer (Agilent Technologies, Tokyo, Japan). Bi was used as the internal standard. The ICP-MS measurements were performed for the supernatant obtained after mixing [Pt_17_(CO)_12_(PPh_3_)_8_]Cl_*n*_ with γ-Al_2_O_3_ to estimate the unadsorbed Pt content. The adsorption efficiency and the Pt amount on γ-Al_2_O_3_ were estimated using this value.

ICP-OES was performed using an Agilent Technologies 700 series spectrometer to determine the Pt content in Pt_17_/γ-Al_2_O_3_ or Pt_NP_/γ-Al_2_O_3_ after completely dissolving the sample using aqua regia.

TG-MS was performed with an STA 2500 Regulus (NETZSCH) and a JMS-Q 1500GC (JEOL) at a heating rate of 5 °C min^−1^ under an Ar atmosphere over the temperature range 25–900 °C using ∼3 mg sample of Pt_17_(CO)_12_(PPh_3_)_8_/γ-Al_2_O_3_.

XPS data were collected using an electron spectrometer (JEOL, JPS-9010MC) equipped with a chamber at a base pressure of ∼2 × 10^−8^ Torr. X-rays from the Mg-Kα line (1253.6 eV) were used for excitation.

Pt L_3_-edge XAFS measurements were performed at beamline BL01B1 of the SPring-8 facility of the Japan Synchrotron Radiation Research Institute (proposal numbers 2018B1422 and 2019A0944). The incident X-ray beam was monochromatized using a Si(111) double-crystal monochromator. As references, the XAFS spectra of Pt foil and solid PtO_2_ were recorded in transmission mode using ionization chambers. The Pt L_3_-edge XAFS spectra of the samples were measured in fluorescence mode using a 19-element Ge solid-state detector at room temperature. The X-ray energies for the Pt L_3_-edges were calibrated using Au foil. The XANES and EXAFS spectra were analyzed using the xTunes program^77^ as follows. The χ spectra were extracted by subtracting the atomic absorption background using cubic spline interpolation and normalized to the edge height. The normalized data were used as the XANES spectra. The *k*^3^-weighted χ spectra in the *k* range 3.0–14.0 Å^−1^ for the Pt L_3_-edge were Fourier transformed into *r* space for structural analysis. The curve fitting analysis was performed in the range of 1.2–3.0 Å for the Pt L_3_-edge. In the curve fitting analysis, the phase shifts and backscattering amplitude function of Pt–C, Pt–P, and Pt–Pt were calculated using the FEFF8.5L program.

### Measurements of catalytic activity

Catalytic activity tests on the honeycomb catalysts of Pt_17_/γ-Al_2_O_3_ or Pt_NP_/γ-Al_2_O_3_ were performed using a flow reactor. The honeycomb catalysts were fixed in a tubular reactor, and their catalytic activity was evaluated by supplying a gas mixture containing a reducing gas (CO or C_3_H_6_), an oxidation gas (O_2_), and a carrier gas (N_2_) at a space velocity (SV) of 50 000 L h^−1^ (Table S4†). The conversions of CO or C_3_H_6_ were monitored using an exhaust gas analyzer (MEXA-ONE-D1, HORIBA) from 100 °C to 400 °C at a heating rate of 20 °C min^−1^. Before each catalytic activity test, the honeycomb catalyst was pre-treated at 400 °C for 0.5 h under a flow of the gas mixture used for the catalytic activity test.

### Aging treatment for durability tests

The aged catalyst samples were prepared by hydrothermal redox aging under a flow of perturbed reduction gas (3% of H_2_, 3% of CO and 10% of H_2_O) and oxidation gas (3% of O_2_ and 10% of H_2_O) with N_2_ balance (Table S5†). The perturbation cycle was for 3 min, the aging temperature was 1000 °C, and the duration was 4 h.

## Author contributions

Y. Negishi and S. Nagaoka designed the experiments and conducted the measurement with the help of N. Shimizu, K. Funai, R. Kaneko, K. Wakamatsu, A. Harasawa, S. Hossain, W. Kurashige and T. Kawawaki. S. Yamazoe conducted XAFS experiments at SPring-8. M. E. Schuster and D. Ozkaya conducted HAADF-STEM experiments in England. Y. Negishi and S. Nagaoka co-wrote the paper and all authors have approved the final version of the manuscript.

## Conflicts of interest

There are no conflicts to declare.

## Supplementary Material

NA-002-C9NA00579J-s001
