# Supplementary material for: γ-Alumina-supported Pt17 cluster: controlled loading, geometrical structure, and size-specific catalytic activity for carbon monoxide and propylene oxidation
Source: Nanoscale Adv. 2019 Dec 3;2(2):669–78. doi: 10.1039/c9na00579j (PMC9417680; doi:10.1039/c9na00579j)
Supplement: NA-002-C9NA00579J-s001 [file NA-002-C9NA00579J-s001.pdf]

Electronic Supplementary Material (ESI) for Nanoscale Advances.
This journal is © The Royal Society of Chemistry 2020

Supporting Information

γ-Alumina-Supported Pt_{17} Cluster: Controlled Loading, Geometrical Structure, and Size-Specific Catalytic Activity for Carbon Monoxide and Propylene Oxidation

Yuichi Negishi[a,b,*], Nobuyuki Shimizu,[a] Kanako Funai,[a] Ryo Kaneko,[a] Kosuke Wakamatsu,[a] Atsuya Harasawa,[a] Sakiat Hossain,[a] Manfred E. Schuster,[c] Dogan Ozkaya,[c] Wataru Kurashige,[d] Tokuhisa Kawawaki,[a,b] Seiji Yamazoe[e,*] and Shuhei Nagaoka[d,*]

[a]*Department of Applied Chemistry, Faculty of Science, Tokyo University of Science, 1−3 Kagurazaka, Shinjuku−ku, Tokyo 162−8601, Japan*

[b]*Photocatalysis International Research Center, Tokyo University of Science, 2641 Yamazaki, Noda, Chiba 278−8510, Japan*

[c]*Johnson Matthey Technology Centre, Blounts Court, Sonning Common, Reading RG4 9NH, UK*

[d]*Johnson Matthey Japan, G.K., 5123−3, Kitsuregawa, Sakura, Tochigi 329−1492, Japan*

[e]*Department of Chemistry, Graduate School of Science, Tokyo Metropolitan University, 1–1 Minami-Osawa, Hachioji-shi, Tokyo 192−0397, Japan.*

Corresponding Author E-mail: negishi@rs.kagu.tus.ac.jp (Y. Negishi), yamazoe@tmu.ac.jp (S. Yamazoe), Shuhei.Nagaoka@mattheyasia.com (S. Nagaoka),

1. Additional Tables

Table S1. Curve Fitting Analysis of Pt L_3-edge EXAFS Data for $[Pt_{17}(CO)_{12}(PPh_3)_8]Cl_n$

Bond	C.N. [a,b]	R(Å) [a]	D.W. [a,c]	R factor (%) [a]
Pt−C	1.6(4)	2.02(6)	0.008(7)	
Pt−P	0.5(2)	2.44(7)	0.004(3)	11.9
Pt−Pt	6.9(6)	2.63(6)	0.020(10)	

The numbers in parentheses are uncertainties; 1.6(4) and 2.02(6) represent 1.6 ± 0.4 and 2.02 ± 0.06, respectively.
[a] These values were obtained by fitting with Pt–C, Pt–P, or Pt–Pt bonds.
[b] Coordination number
[c] Debye–Waller factor.

Table S2. Curve Fitting Analysis of Pt L_3-edge EXAFS Data for $Pt_{17}(CO)_{12}(PPh_3)_8$/γ-$Al_2O_3$

Bond	C.N. [a,b]	R(Å) [a]	D.W. [a,c]	R factor (%) [a]
Pt−C	1.5(2)	2.01(4)	0.003(2)	
Pt−P	0.4(2)	2.24(6)	0.004(3)	15.0
Pt−Pt	5.0(4)	2.59(4)	0.013(6)	

The numbers in parentheses are uncertainties; 1.5(2) and 2.01(4) represent 1.5 ± 0.2 and 2.01 ± 0.04, respectively.
[a] These values were obtained by fitting with Pt–C, Pt–P, or Pt–Pt bonds.
[b] Coordination number
[c] Debye–Waller factor.

Table S3. Curve Fitting Analysis of Pt L_3-edge EXAFS Data for Pt_{17}/γ-Al_2O_3

Bond	C.N. [a,b]	R(Å) [a]	D.W. [a,c]	R factor (%) [a]
Pt−C	3.5(3)	2.03(4)	0.006(4)	10.0
Pt−Pt	6.6(3)	2.76(3)	0.009(4)	

The numbers in parentheses are uncertainties; 3.5(3) and 2.03(4) represent 3.5 ± 0.3 and 2.03 ± 0.04, respectively.
[a] These values were obtained by fitting with Pt–C or Pt–Pt bonds.
[b] Coordination number
[c] Debye–Waller factor.

Table S4. Gases Used in Oxidation Reaction of CO and C_3H_6

Reaction	CO/C_3H_6	O_2	N_2
CO oxidation	1%	0.5%	98.5%
C_3H_6 oxidation	200 ppm	0.5%	~99.5%

Table S5. Gases Used in Aging Treatment

Atmosphere	H_2	CO	O_2	H_2O	N_2
Oxidation	0%	0%	3%	10%	87%
Reduction	3%	3%	0%	10%	84%

2. Additional Schemes

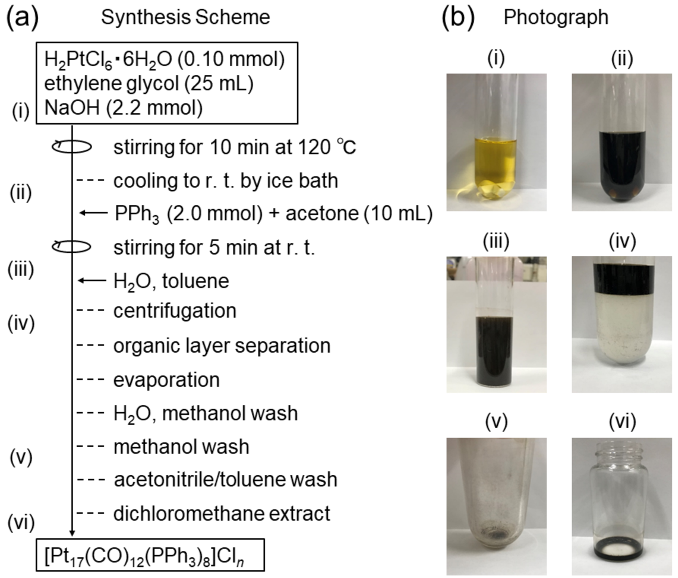

Scheme S1. (a) Synthesis procedure for $[Pt_{17}(CO)_{12}(PPh_3)_8]Cl_n$ (n = 1, 2) and (b) photograph of product at each stage (i)–(vi) described in (a).[1]

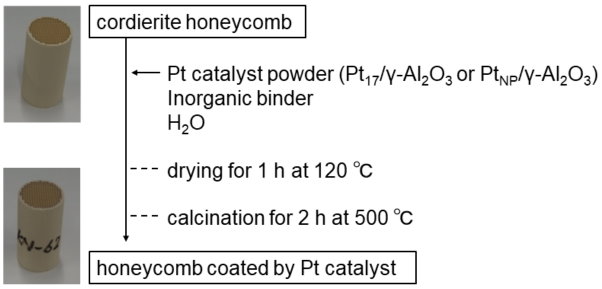

Scheme S2. Preparation procedure for honeycomb catalysts.

3. Additional Figures

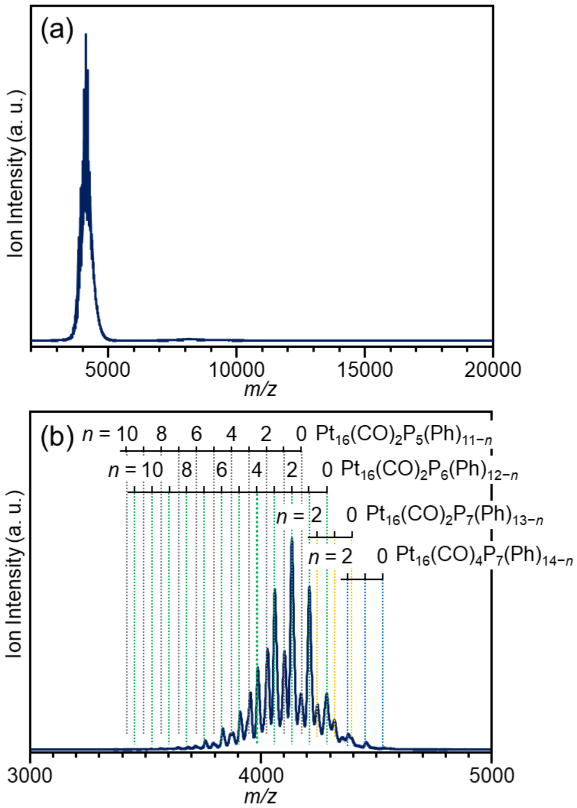

Figure S1. Positive-ion MALDI mass spectra: (a) wide-region spectrum and (b) spectrum expanded for the main peaks. These mass spectra include the laser fragments assigned in (b). In (a), peaks other than the fragment peaks of $[Pt_{17}(CO)_{12}(PPh_3)_8]Cl_n$ are hardly observed, indicating that the product contains high-purity $[Pt_{17}(CO)_{12}(PPh_3)_8]Cl_n$.

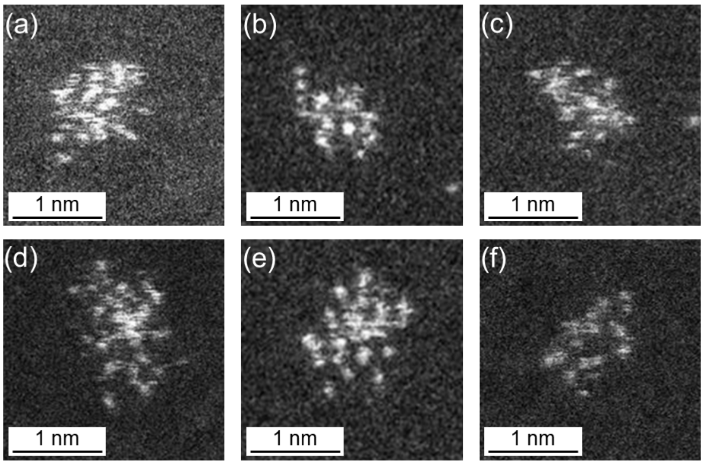

Figure S2. (a)–(f) Representative HAADF-STEM images of $[Pt_{17}(CO)_{12}(PPh_3)_8]Cl_n$.

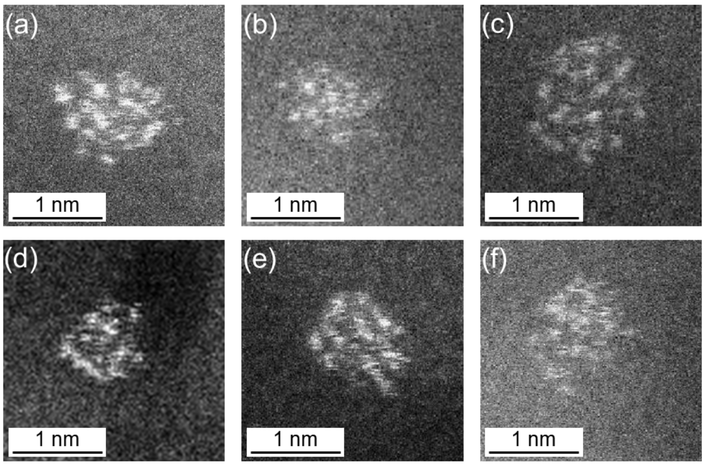

Figure S3. (a)–(f) Representative HAADF-STEM images of $Pt_{17}(CO)_{12}(PPh_3)_8/\gamma\text{-}Al_2O_3$.

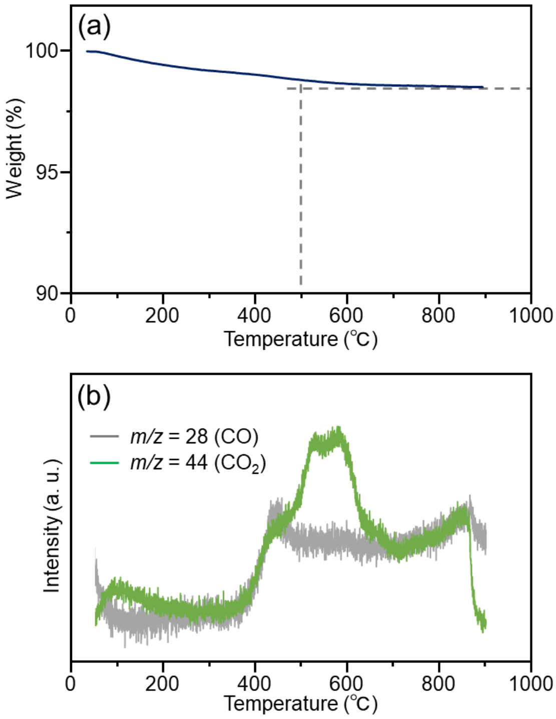

Figure S4. (a) TGA curve obtained for $Pt_{17}(CO)_{12}(PPh_3)_8/\gamma\text{-}Al_2O_3$ and (b) the gasses desorbed from the sample above 400 °C. These curves were obtained using an STA 2500 Regulus (NETZSCH) and a JMS-Q 1500GC (JEOL) at a heating rate of 5 °C/min under Ar atmosphere over the temperature range 25−900 °C. In (b), CO_2 is considered to be the product of the oxidation of the CO ligand catalyzed by Pt_{17} because this measurement was conducted under Ar atmosphere.[2] These results imply that some of the CO remains on the supported Pt_{17} even after the calcination at 500 °C.

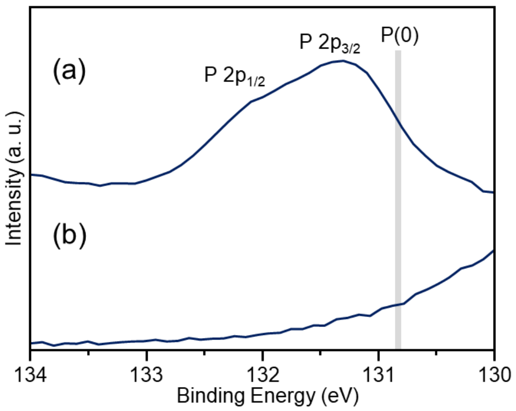

Figure S5. P 2p XPS spectra of (a) $[Pt_{17}(CO)_{12}(PPh_3)_8]Cl_n$ and (b) $Pt_{17}/\gamma\text{-}Al_2O_3$.

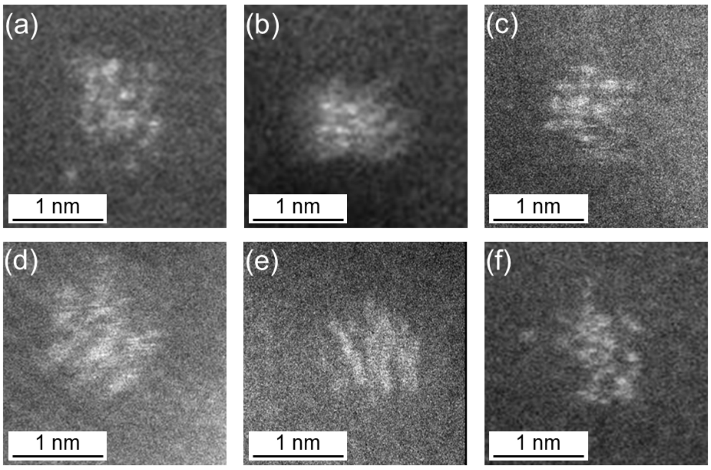

Figure S6. (a)–(f) Representative HAADF-STEM images of $Pt_{17}/\gamma\text{-}Al_2O_3$.

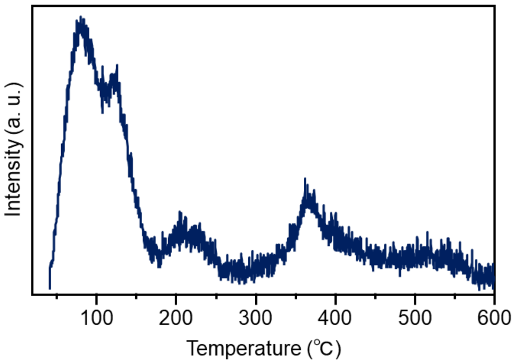

Figure S7. Temperature-programmed reaction (TPR) curve monitored at m/z = 44 (CO_2) for $Pt_{17}/\gamma\text{-}Al_2O_3$ sample after air exposure. TPR analysis was performed with a Rigaku TPD type R analyzer at a heating rate of 20 °C/min under a flow of 10% O_2 diluted in He using ~100-mg samples of the catalyst powders.

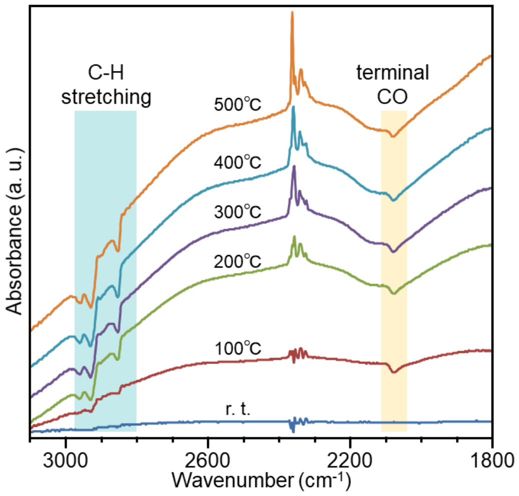

Figure S8. Monitoring of the desorbed gases from $Pt_{17}/\gamma\text{-}Al_2O_3$ at each temperature using FT-IR spectroscopy. This experiment was conducted using FT/IR-6600 spectrometer (JASCO) with KP1000 digital program controller (CHINO) under a flow of 10% O_2 diluted in He. These spectra were obtained by subtracting the room-temperature spectrum from the spectrum of each temperature (100−500 °C); thus, the peaks originating from the desorbed species appear under the base line. These spectra imply that the CO adsorbed on $Pt_{17}/\gamma\text{-}Al_2O_3$ is related to the CO_2 observed in the TPR curve (Figure S7).

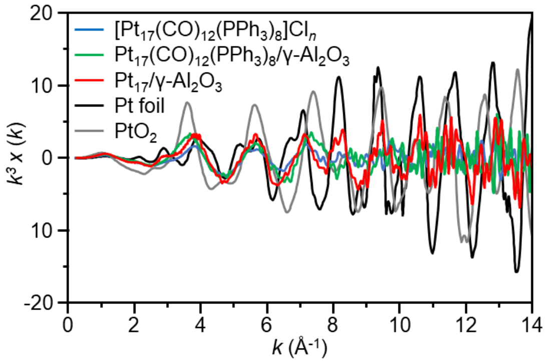

Figure S9. Pt L_3-edge EXAFS spectra of $[Pt_{17}(CO)_{12}(PPh_3)_8]Cl_n$, $Pt_{17}(CO)_{12}(PPh_3)_8/\gamma\text{-}Al_2O_3$, and $Pt_{17}/\gamma\text{-}Al_2O_3$ together with those of Pt foil and PtO_2 for comparison.

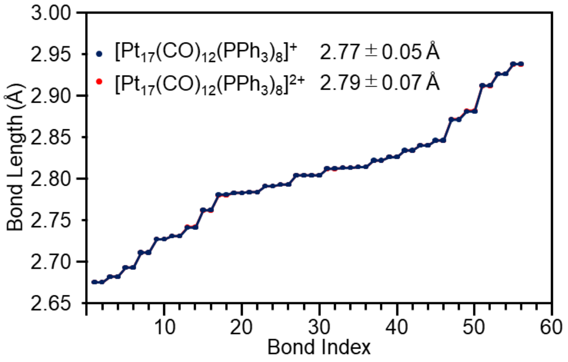

Figure S10. Pt−Pt bond lengths of $[Pt_{17}(CO)_{12}(PPh_3)_8]^+$ (blue) and $[Pt_{17}(CO)_{12}(PPh_3)_8]^{2+}$ (red) estimated from each geometrical structure reported in our previous paper[1].

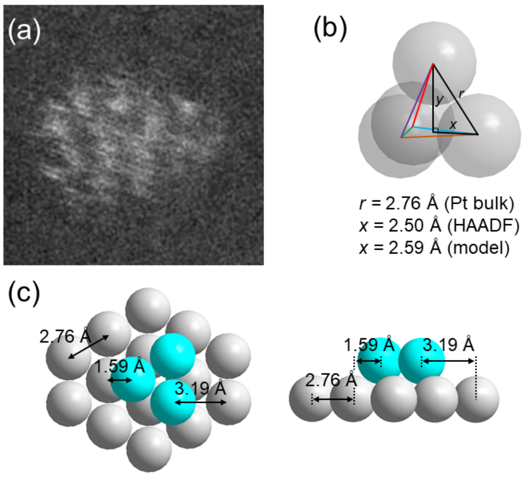

Figure S11. Estimation of model structures of $Pt_{17}/\gamma\text{-}Al_2O_3$ for bi-layered structure: (a) HAADF-STEM image, (b) method to arrange Pt atoms, and (c) proposed bi-layered structure and estimated bond lengths for this structure.

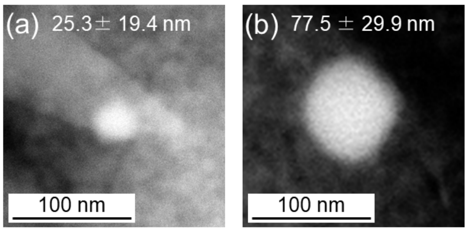

Figure S12. HAADF-STEM images of (a) $Pt_{17}/\gamma\text{-}Al_2O_3$ and (b) $Pt_{NP}/\gamma\text{-}Al_2O_3$ after aging treatment.

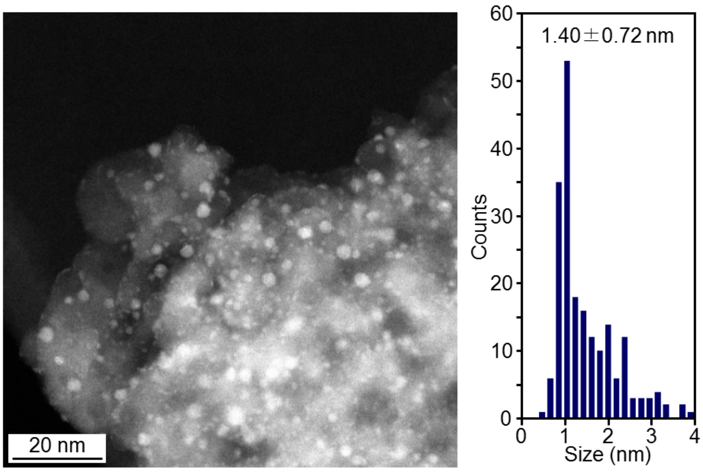

Figure S13. HAADF-STEM images of $Pt_{17}/\gamma\text{-}Al_2O_3$ loaded with a weight of 0.7 wt% Pt. The aggregation of Pt_{17} clusters were not necessarily suppressed at this loading weight, although the size distribution is still narrow (1.40 ± 0.72 nm) compared with that of Pt_{NP} prepared using the conventional method with lower loading weight (0.15% Pt; 3.10 ± 3.14 nm). In order to achieve the higher loading weight, we need to modify the ligand of Pt_{17} clusters or increase the surface defects of $\gamma\text{-}Al_2O_3$ to suppress the aggregation on the $\gamma\text{-}Al_2O_3$ during the calcination.

4. References

1. L. V. Nair, S. Hossain, S. Wakayama, S. Takagi, M. Yoshioka, J. Maekawa, A. Harasawa, B. Kumar, Y. Niihori, W. Kurashige and Y. Negishi, *J. Phys. Chem. C,* 2017, **121**, 11002−11009.
2. D. Gavril, V. Loukopoulos and G. Karaiskakis, *Chromatographia*, 2004, **59**, 721−728.
